# Advances in Dysprosium Recovery from Secondary Sources: A Review of Hydrometallurgical, Biohydrometallurgical and Solvometallurgical Approaches

**DOI:** 10.3390/molecules31010176

**Published:** 2026-01-02

**Authors:** Ewa Rudnik

**Affiliations:** Faculty of Non–Ferrous Metals, AGH University of Krakow, Al. Mickiewicza 30, 30-059 Krakow, Poland; erudnik@agh.edu.pl

**Keywords:** dysprosium, leaching, separation techniques, neodymium magnets, phosphogypsum, coal ash

## Abstract

Dysprosium is one of the most critical elements for global economies due to its essential role in the green energy transition. Although it is added in small quantities as an alloying element, dysprosium plays a crucial role in NdFeB magnets used in wind turbines and industrial motors. On the other hand, the limited resources and production capacity of dysprosium contribute to supply shortages and raise concerns about its long-term availability. Therefore, there is a need for efficient techniques that will enable the recovery of dysprosium from secondary materials to bridge the gap between supply and demand while addressing the risks associated with securing a stable supply. This review focuses on (bio)hydrometallurgical and solvometallurgical methods for recovering dysprosium from key secondary sources such as spent NdFeB magnets, phosphogypsum, and coal ash. Although these wastes do not always contain high concentrations of dysprosium, they can have a simpler elemental composition compared to primary sources (a few tens or hundreds of ppm Dy) and are more readily available. Spent NdFeB magnets, with a few percent Dy, show the most promise for recycling. In contrast, coal fly ashes (with several ppm Dy), although widely available, bind dysprosium in an inert phase, requiring substantial pretreatment to enhance the release of the desired element. Phosphogypsum, while not yet a significant source of dysprosium (several ppm Dy), is increasingly recognized as a potential source for other rare earth elements. Although conventional hydrometallurgical methods are commonly used, these are typically unselective for dysprosium recovery, whereas unconventional solvometallurgical approaches show preferential extraction of dysprosium over base metals.

## 1. Introduction

**Figure 1:**
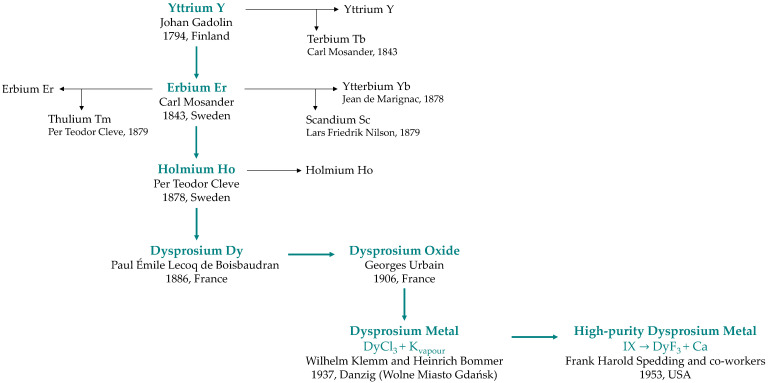
Genealogy of dysprosium discovery and metal isolation.

Dysprosium is one of the more intriguing members of the heavy rare-earth subgroup. Its name derives from the Greek word *dysprositos* (“hard to obtain”), a description that aptly reflects the element’s complex history (Figure 1), from its spectral discovery [1], the first isolation of the oxide [2] and metallic form [3], to the production of high-purity metal achieved only after the development of ion-exchange technique [4] and metallothermic reduction [5].

Dysprosium is a silvery-gray metal with a density of 8.55 g/cm^3^ [6]. It is relatively hard (Vickers hardness of 410–550 MPa), but can be cut with a knife. It is malleable, capable of being rolled to about a 30% reduction in cross-section without heat treatment, and can be machined without sparking. Its high melting (1412 °C) and boiling (2567 °C) points, combined with a very large thermal neutron absorption cross-section (around 2200–2900 barns for the ^164^Dy isotope [7,8]), make dysprosium ideal for alloying with special stainless steels for control applications in nuclear reactors.

Dysprosium is paramagnetic at room temperature, but upon cooling, it becomes antiferromagnetic below the Néel temperature of about −97 °C, and then ferromagnetic below the Curie temperature of about −186 °C [6]. It also exhibits several modulated magnetic phases, giving rise to a rich magnetic phase diagram [9]. Dysprosium shows the highest effective magnetic moment among naturally occurring elements, comparable only to holmium (10.6 μ_B_/atom at 25 °C) [6]. The uncommon magnetic properties of dysprosium were promptly recognized [10] and exploited in the 1970s (USA) for the development of Terfenol-D, Tb_x_Dy_1−x_Fe_2_ (x ≈ 0.3) [11]. This alloy exhibits the highest magnetostrictive performance among all known alloys [12], characterized by an electromechanical coupling coefficient of 0.73, giant magnetostriction of 800–1600 ppm, rapid response time, low magnetic anisotropy at room temperature and high energy density. In this material, the dysprosium component facilitates induction magnetoresistive responses (i.e., expansion and contraction) at lower magnetic field intensities. These properties make Terfenol-D suitable for a variety of applications, including sonar systems, magneto-mechanical sensors, actuators, and ultrasonic sensors and transducers, as well as in precision positioning and machining, fuel injectors, inkjet printers, motors, pumps, valves, brakes, etc.

**Figure 2:**
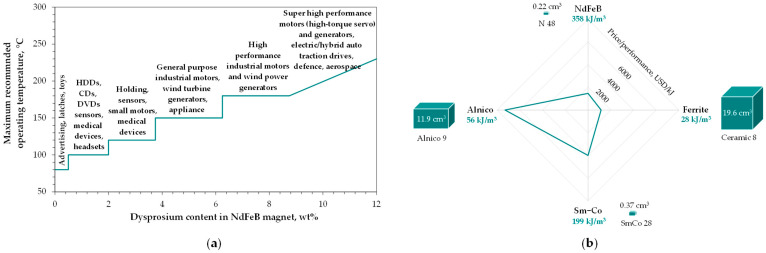
Main applications of dysprosium-containing NdFeB magnets (**a**) and their price-to-performance ratio (with maximum energy product and magnet size to generate a 0.1 T magnetic field at 5 mm from the pole face) compared to other commercially available permanent magnets (**b**). Based on [13,16].

Undoubtedly, the most common utilization of dysprosium’s magnetic properties is its essential function in NdFeB magnets, a breakthrough recognized in the 1980s (Japan, USA) [13]. Partial substitution of neodymium with dysprosium in these materials improves their performance by resisting demagnetization at operating temperatures of 150–200 °C [13,14] and enhancing corrosion resistance [15]. This development revolutionized the commercial applications of neodymium magnets (Figure 2a), as they offer the highest potential magnetostatic energy per unit volume (near 360 kJ/m^3^) at room temperature among all permanent magnets available on the market (Figure 2b), placing them second only to the most affordable ferrite magnets in terms of the price-to-performance ratio [16].

**Figure 3:**
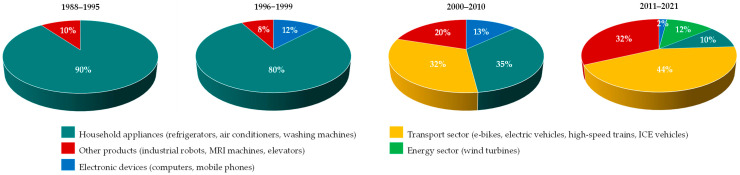
Global changes in the end uses of dysprosium between 1988 and 2021. Based on [22].

The NdFeB magnets dominate the permanent magnet market, accounting for 58% of global share in 2024 [17]. Their importance continues to rise, driven primarily by high-tech sectors such as electric vehicles and wind power generation due to the gradual shift away from fossil-fuelled vehicles and the global transition toward green energy [18,19]. As a result, the applications of dysprosium have evolved over time from household appliances to advanced technologies (Figure 3). This has also impacted the demand for dysprosium [20,21], which has been progressively increasing over the past decades, reflecting a rise in global annual dysprosium mine production, which reached 2000–3000 tonnes (as oxide) in 2020s [22,23], compared to 135–185 tonnes in the early 1990s [22]. In line with this trend, market forecasts indicate that the value of the dysprosium sector will grow from USD 951.849 million in 2025 to USD 1305.285 million by 2030, representing a CAGR of 6.5% [24].

Dysprosium is not as sporadic in nature as the name of its rare-earth group might suggest. Its average concentration in the bulk continental crust is about 3.6 ppm, which is significantly higher than that of truly rare elements such as the platinum-group metals (0.03–1.5 ppb), rhenium (0.19 ppb), gold (1.3 ppb), and even silver (0.06 ppm), indium (0.05 ppm), or mercury (0.03 ppm) [25]. Among the 17 rare earth elements (lanthanides together with yttrium and scandium), dysprosium ranks tenth in abundance in the Earth’s crust.

Although rare-earth ores have been identified on every continent [26], deposits enriched in heavy lanthanides are far less common than those dominated by the light subgroup [27,28]. Dysprosium is sourced primarily from carbonatite-hosted deposits containing monazite-(Ce) (Ce,La,Nd,Th)PO_4_, bastnäsite-(Ce) (Ce,La)(CO_3_)F, and xenotime-(Y) YPO_4_, but it can also be extracted from ion-adsorption clays or eudialyte Na_4_(Ca,REE)_2_(Fe^2+^,Mn,Y)ZrSi_8_O_22_(OH,Cl)_2_. The concentrations of dysprosium are low (Table 1), which limits economically viable extraction in many regions, and in practice only a few countries are able to supply this element to the global market [21,22].

China is the world’s leading producer of dysprosium, with both the largest production capacity and the largest global reserves of heavy rare earth elements (80% [39]), including dysprosium (63% [22]). About 85–90% of all Chinese dysprosium concentrates come from ion-adsorption clay deposits [31,40,41], primarily located in the southern provinces (Jiangxi, Guangdong, Fujian, Hunan, Guangxi) [40,42]. These deposits contain dysprosium concentrations (1.8–7.5%) more than ten times higher than those in monazite or bastnäsite (0.01–0.22%) [43], with the extraction process being significantly easier [39,44]. China’s dominant position has been further strengthened by substantial imports of concentrates from Myanmar, Malaysia, and Australia [31]. Notably, neighboring Myanmar hosts dysprosium-rich (0.06–0.12% Dy) ion-adsorption clay deposits in Kachin and Shan States [45].

**Figure 4:**
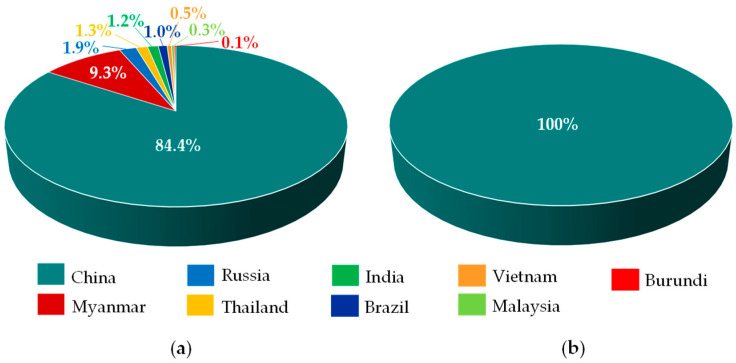
Global shares of dysprosium extraction (**a**) and processing (**b**). Based on [51].

In light of this, it is clear that the concentration of both mining and production in essentially one world region (Figure 4), compounded by China’s export restrictions on seven heavy rare earth elements (Sm, Gd, Tb, Dy, Lu, Sc, Y) since 4 April 2025 [46], has created significant global challenges, particularly regarding availability of the element for key sectors (defense, electronics, automotive, energy) [46,47,48]. This also led to a temporary trading suspension from April to May 2025 [49]. However, current prices (454 USD/kg Dy_2_O_3_ as of 12 December 2025) are significantly lower than the price spikes of 2022 (770 USD/kg Dy_2_O_3_ on 1 January 2022) [49] or the record highs of 2011 (1500 USD/kg Dy_2_O_3_ on average) [50].

**Figure 5:**
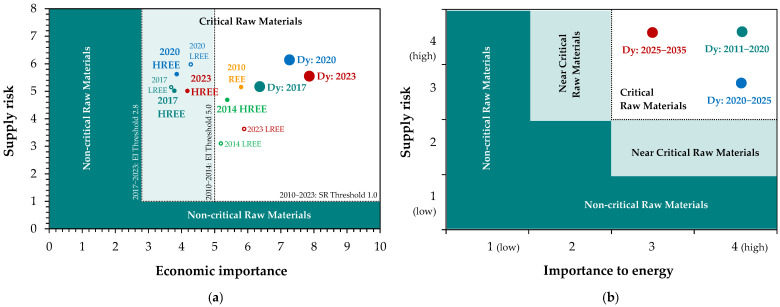
Evolution of the dysprosium position in the criticality matrices of: (**a**) the European Union, compared to REE as groups (based on [51,53,54,55,56]), (**b**) the United States (based on [57,58]).

The demand for dysprosium, combined with its complicated hydrometallurgical separation procedures [39,43] and uncertain supply chains, makes this element particularly critical and strategic for the 21st century [52]. Its significance extends not only to the European Union [51,53,54,55,56] (Figure 5a) and the United States [57,58,59] (Figure 5b), but also to countries like Australia, Brazil, Canada, China, Indonesia, Japan, South Africa, among others [60,61].

**Figure 6:**
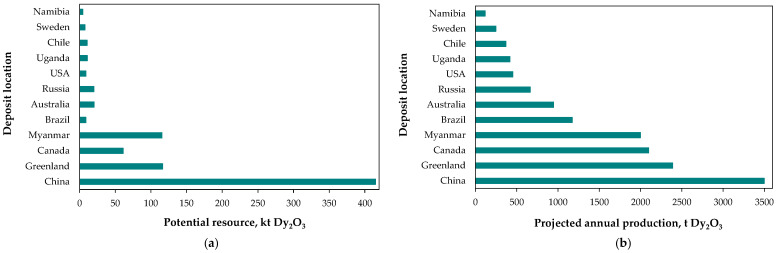
Estimated potential dysprosium resources (**a**) and corresponding annual production forecasts (**b**) for selected projects (about 20) at various stages of development. Adapted from [63].

These circumstances have prompted the search for new natural resources of rare earths, particularly from the heavy subgroup. Among the numerous worldwide projects [27,28,34,62,63,64,65], dysprosium-rich resources have been reported in, among others, South Africa (Zandkopsdrift: 0.77% Dy_2_O_3_ of TREO [34]), Namibia (Lofdal: above 0.07% Dy [64]), Canada (Strange Lake: 4.1% Dy_2_O_3_ of TREO [34]), Sweden (Norre Kärr: 0.027% Dy_2_O_3_ of TREO [37]), Greenland (Kvanefjeld: 1.1% Dy_2_O_3_ of TREO [34]; Kujalleg: 2.9% Dy_2_O_3_ of TREO [28]), and Australia (North Stanmore: 0.022% Dy_2_O_3_ [65]). These efforts demonstrate the high potential for future dysprosium supply (Figure 6), helping to bridge the gap between supply and demand [19]. On the other hand, first dysprosium oxide production operations were launched outside China, with Australian Lynas Rare Earths at its Malaysian facility (May 2025) [66] and American Energy Fuels at its White Mesa Mill in Utah (August 2025) [67].

While primary sources of dysprosium play a key role, secondary resources should not be overlooked. These include mainly NdFeB magnet scraps, phosphate-type tailings, as well as less conventional ones like coal combustion ashes. In China, neodymium magnet scraps account for about 8–12% of dysprosium production [31,40,41], whereas in Western countries the recycling of rare earths (including dysprosium) is practically nonexistent (below 1% in the EU) [19,30,62]. As a result, different demand scenarios have predicted a global dysprosium shortage for years, even with the implementation of its recycling [34,41,61,68,69,70]. This highlights the need to pay more attention to this issue. Therefore, the goal of this work is to explore advanced, cost-effective (bio)hydrometallurgical and solvometallurgical methods [71] for recovering dysprosium from various secondary materials, emphasizing their importance in sustainable global economy and green transformation [72]. This is especially noteworthy in view of the environmental concerns and accidents that have arisen during the extraction of rare earths from natural deposits [40,45,62].

## 2. Dysprosium Recovery from Waste Permanent Magnets

### 2.1. General Characterization

Neodymium permanent magnets represent the principal secondary raw materials for dysprosium recovery. This category includes both magnet swarf generated during manufacturing and spent magnets reclaimed from end-of-life products (e.g., consumer electronics, hard disc drives, motors, wind turbine generators). They offer substantial potential, not only due to their large material volume and availability [21,22], but also higher concentrations of dysprosium (about 4% on average [73,74]) and simpler composition compared to concentrates from primary sources (Table 2).

The current global production capacity for NdFeB permanent magnets is approximately 240,000 tonnes per year [79], with an increasing trend driven by rising demand and the commissioning of new plants [80]. Since the lifespan of NdFeB magnets ranges from 2 to 3 years in consumer electronics to 20–30 years in wind turbines [81,82], the potential for secondary dysprosium sources develops over time. Du and Graedel [82] estimated that, the in-use stock of neodymium magnets (in 2007) contained around 16,000 tonnes of recoverable dysprosium. In turn, Reimer et al. [83] projected that the realistic potential return of magnets accumulated between 2018 and 2040 in the EU would be around 25,000 tonnes, corresponding to about 1100 tonnes of secondary dysprosium.

### 2.2. Pretreatment Stages

**Figure 7:**
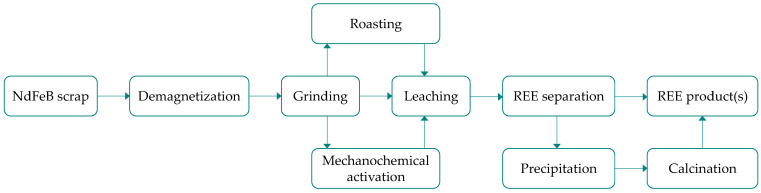
Typical procedures of REE recovery from waste NdFeB magnets via leaching stage.

Chemical treatment of waste NdFeB magnets in liquid leachants often requires preliminary processing (Figure 7). This typically involves reducing the particle size to tens or hundreds of micrometers through grinding [84,85,86,87,88] to increase the surface area available for heterogeneous reactions and enhance reaction kinetics. Romano et al. [85] observed that as the particle size of the waste powdered material decreased from 1000–1700 μm to below 40 μm, the dysprosium content increased gradually from 1.97% to 3.64%. Notably, Belfqueh et al. [84] reported that exposure of the magnet to oxygen during grinding induces structural changes in the material, leading to the formation of oxides, including the mixed oxide NdFeO_3_, which is problematic for leaching under mild conditions. Therefore, an inert (nitrogen) atmosphere was recommended not only to preserve composition of the hard magnetic phase (Nd_2_Fe_14_B) but also for safety reasons, to prevent self-ignition. Similar phenomena were observed during thermal treatment in air [89] applied to demagnetize the waste magnets (typically at 350 °C [84,88]). Different roasting procedures can be used to convert metallic phases into more leachable compounds [88]. Mechanochemical was also investigated [90], although no data on dysprosium behavior were reported in such studies. The effects of the pretreatment stages on the leachability of the waste magnets are discussed in subsequent chapters.

### 2.3. Hydrometallurgical Treatment

Hydrometallurgical treatment of spent NdFeB magnets generally involves acid leaching followed by the separation of REE metals from iron ions through solvent extraction (Table 3). Both inorganic [88,89,91,92,93,94] and organic [84,86,87,95] acids can efficiently dissolve the metals, although the process is not highly selective and does not ensure element separation at the initial stage:

Dy + 3H^+^ → Dy^3+^ + 1½H_2_ (1)

Sun et al. [89] compared the leaching efficiency of magnet components in various mineral acids (HCl, HNO_3_, H_2_SO_4_, H_3_PO_4_), paying particular attention to the behavior of neodymium and dysprosium. They observed that dysprosium can be almost completely extracted in HCl, HNO_3_, and H_2_SO_4_ (1 M, 70 °C, 2 h), while the process was completely ineffective in H_3_PO_4_. Notably, neodymium could be fully extracted only in HNO_3_, whereas in the remaining acids the extraction efficiency did not exceed 40%.

In turn, Belfqueh et al. [84] evaluated the applicability of four organic acids: acetic, formic, citric, and tartaric (up to ~15 M) as green leachants. Although the process was not selective toward REEs, high leaching efficiencies over a wide range of solid-to-liquid ratios (up to 8%) were obtained only with acetic acid. On the other hand, at low acid concentrations, secondary precipitation of iron in the form of goethite was observed. Gergoric et al. [95] demonstrated similar performance of citric and acetic acids (1 M) in achieving complete Dy extraction at room temperature from air-roasted NdFeB. In both cases, a positive effect of increasing acid concentration (0.1–1 M) was observed. More detailed studies on leaching with citric acid were carried out by Romano et al. [85,87]. They showed that increasing the leachant concentration from 0.5 M to 2.5 M raised the dysprosium recovery from about 63% to about 94% (6 h), with an optimal value of 1.45 M being selected (92% yield). They also applied a shrinking-core model, indicating internal and/or film diffusion as the rate-controlling steps of dysprosium leaching.

Alternatively, water can be used as the cheapest leachant. However, its applicability is restricted to NdFeB magnets that have undergone prior chloridizing [94] or sulfating [96] roasting:

2Dy + 6NH_4_Cl + 1½O_2_ → 2DyCl_3_ + 6NH_3_ + 3H_2_O (2)

2Dy + 6H_2_SO_4_ → Dy_2_(SO_4_)_3_ + 3SO_2_ + 6H_2_O (3)

Importantly, only the latter pretreatment proved effective for dysprosium recovery due to high solubility of REE sulfates at lower temperatures. It is also noteworthy that chloridizing roasting may lead to the formation of lanthanide oxychlorides, which are poorly soluble in water, while their dissolution proceeds much more effectively in HCl solutions [88,94]:

DyCl_3_ + ½O_2_ → DyOCl + Cl_2_ (4)

DyOCl + 2HCl → DyCl_3_ + H_2_O (5)

Nonetheless, significant improvement in the conversion of REEs into soluble chlorides can be achieved when a large excess of the chloridizing agent is used; for example, almost complete dysprosium extraction was obtained at an NH_4_Cl:NdFeB ratio of 5 during roasting stage [94].

The most problematic step in treating the leach liquor is the separation REEs of low-concentration from other elements, primarily iron. This step is generally carried out using solvent extraction [89,92,95], precipitation of REE oxalates [88,94], or a combination of both methods [91]. Effective extractants include D2EHPA [86,95], TOPO [89], and Cyanex 302 [92]. However, other studies using synthetic solutions have shown high dysprosiumseparation potential of PC 88A [97], Cyanex 272 [97,98], TBP [97], Cyanex 572 [99], INET-3 [100] as examples. While solvent extraction allows the separation of dysprosium ions from other REEs, precipitation with oxalic acid produces a mixture of oxalates:

2REE^3+^ + 3H_2_C_2_O_4_ → REE_2_(C_2_O_4_)_3_↓ + 6H^+^ (6)

These are then calcined to form a final oxide mixture with relatively low Dy_2_O_3_ contents (Table 3):

REE_2_(C_2_O_4_)_3_ → REE_2_O_3_ + 3CO_2_ ↑+ 3CO↑ (7)

It should be emphasized that the sequence of metal extraction changes completely when ionic liquids are used as extractants [101,102] and, in contrast to conventional systems, ionic liquids preferentially transfer REE ions rather than transition metals such as iron or cobalt from the aqueous phase. Riaño and Binnemans [101] demonstrated that dysprosium and neodymium ions can be extracted at similarly high levels (90–95%) from a synthetic nitrate solution using trihexyl(tetradecyl)phosphonium nitrate, while cobalt ions remain in the aqueous phase. Further separation of dysprosium can be achieved by stripping Dy^3+^ with an 10M NH_4_NO_3_–EDTA solution. Subsequent oxalate precipitation yields Dy_2_O_3_ with a final purity of 99.8% and a recovery efficiency of 99.1% under optimized conditions. In turn, Wu et al. [102] reported selective extraction of dysprosium, reaching levels of up to 90% from an synthetic acidic chloride solution using the ionic liquid [methyltrioctylammonium][o-octyloxybenzoic acetate] ([N_1888_][OOB]). Both efficiency and selectivity (from Nd, Pr, Fe, Co) increased as the pH was raised from 2.0 to 2.9. This approach is particularly notable because stripping stage can be performed using only dilute HCl solutions (10 mM). Recently, Das et al. [103] proposed the use of the ionic liquid [P_66614_][Cy272] for REE separation from a real acidic chloride leachate obtained from NdFeB magnets. In their approach, the solvent extraction step was preceded by iron removal through precipitation as hematite Fe_2_O_3_. Despite this additional pretreatment, the ionic liquid still exhibited preferential extraction of Dy^3+^ ions, reaching about 75%, whereas the yields for Nd^3+^ and Pr^3+^ were about 30%.

Alternative routes for dysprosium separation from NdFeB magnet leachates (usually synthetic solutions) involve adsorption processes [104]. These use a variety of different adsorbents, with more or less complicated preparation procedures, for example, vermiculite clay [105], zirconium phosphate [106,107], phosphate-functionalized sodium alginate hydrogel [108], functionalized silica [109], pyrolyzed spent tire rubber [110]. However, none of the adsorbents show selectivity for the separation of Dy^3+^ and Nd^3+^ ions, although the efficiency of adsorption–desorption depends on the type of solution used. Undoubtedly, a major advantage of these adsorbents is their low cost, high adsorption capacity, ease of process implementation, and, most importantly, the ability to concentrate ions, thus enabling effective recovery from highly diluted solutions.

**Figure 8:**
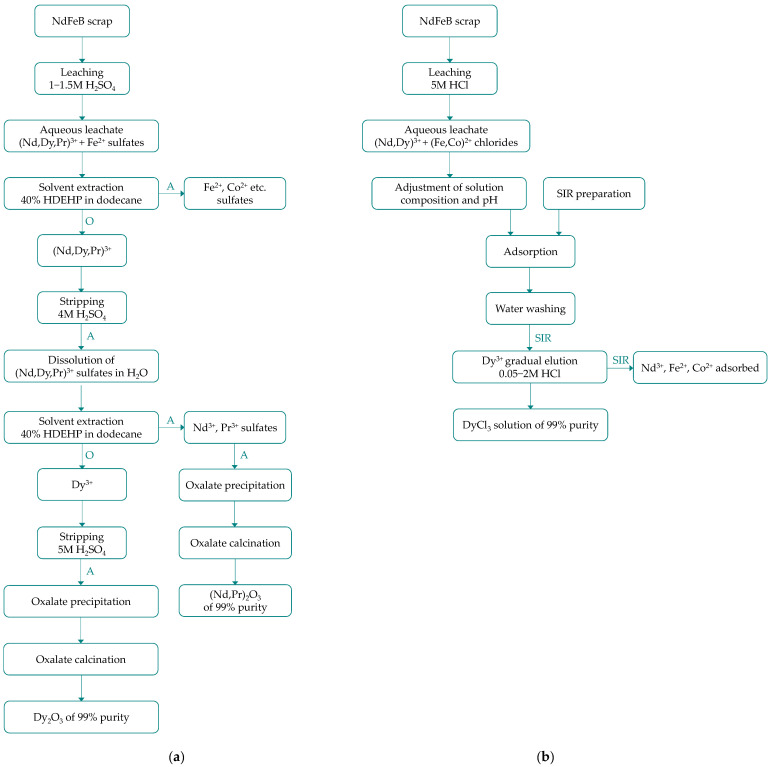
Schemes of hydrometallurgical dysprosium recovery from spent NdFeB magnets: (**a**) with SX separation step (based on [91]); (**b**) with hybrid SIR separation step (based on [93]).

Complete recovery procedures for high-purity dysprosium from spent NdFeB magnets have also been developed [91,93]. Gruber and Carsky [91] carried out a pilot-scale process in three main steps (Figure 8a). The first step involves leaching the magnets in 1–1.5M H_2_SO_4_. As the process generates reactive BH_3_ and H_2_ gases, they must be safely captured in the off-gas stream. However, these gases also promote the reduction of iron ions to Fe(II), which are not transferred to the organic phase during the subsequent liquid–liquid extraction. The solvent extraction is performed using bis-(2-ethylhexyl)phosphoric acid HDEHP in two stages. In the first step, all lanthanides (Dy, Nd, Pr) are extracted from the acidic sulfate aqueous solution, leaving iron species in the raffinate. The lanthanides are then stripped from the organic phase using 4M H_2_SO_4_, resulting in the formation of lanthanide sulfate crystals. These crystals are dissolved in a large volume of water and subjected again to solvent extraction with HDEHP in a counter-current vibrating plate extraction column. This step selectively extracts dysprosium into the organic phase, which is then stripped with 5M H_2_SO_4_. The final dysprosium oxide product, with a purity of 99%, is obtained by precipitation of dysprosium oxalate followed by calcination.

Recently, Yoshida et al. [93] reported the separation of dysprosium from neodymium, iron and cobalt ions using a solvent-impregnated resin SIR, which combines ion-exchange and solvent extraction behavior. Specifically, they prepared a solid polymer resin HP2MG loaded with a liquid extractant PC 88A. The NdFeB magnets were leached with 5 M HCl. After adjusting the pH to 2.0 and adding ascorbic acid to reduce Fe^3+^, the aqueous leachate was directed onto an adsorption column filled with SIR. The solution flow through the column was maintained until saturation, then the remaining solution was removed using deionized water. Selective dysprosium recovery was achieved by gradient elution with HCl solutions of increasing concentration, from 0.05 M to 2 M. In a connected two-column system, 97% recovery of dysprosium was achieved with 99% purity (Figure 8b). Noteworthy, this approach was a simplified route of the earlier procedure developed for the dysprosium separation from sulfate magnet leachate (leaching with H_2_SO_4_, oxalate precipitation, calcination, oxide dissolution in HNO_3_, SIR column separation) [111].

Finally, it is worth highlighting the unconventional ternary water-organic leaching mixture developed by El Maangar et al. [112]. This mixture consisted of water, sodium salicylate, and ethyl acetate in varying ratios, yet forming a single phase at room temperature. Unlike typical aqueous acid solutions, it promoted the dissolution of dysprosium from NdFeB powder (2.8% Dy), achieving extraction efficiencies of 95–98%, while only 20% of Nd and 1–2% of Fe were leached. This marks a significant improvement over leaching in 2 M acids (HNO_3_, H_2_SO_4_, or HCl) under identical experimental conditions, where no selectivity was observed and extraction levels for all magnet components ranged from 85 to 100, the optimal leaching mixture (21% ethyl acetate, 39% sodium salicylate, and 40% water) ensured highly selective recovery of nearly all dysprosium at temperatures as low as 20–40 °C (S/L 2%). Interestingly, increasing the temperature negatively affected dysprosium extraction but enhanced the recovery of neodymium (75–83%) and iron (up to 25%). Extending the leaching time to 30 h did not yield any improvement, as a stable recovery level was reached within 10 h. Dysprosium recovery from the leaching liquor was accomplished using the classical method of oxalate precipitation followed by calcination, with the final oxide predicted to have a purity of 99.4%. Neodymium and other REEs accumulating in the leaching residue can be recovered through a second stage of leaching, followed by oxalate precipitation of the mixed oxide, while practically all iron remains unprocessed as solid waste. These promising results demonstrate the potential of this ternary system, where sodium salicylate acts as a hydrotrope, enhancing the solubility of ethyl acetate in water. This innovative system not only opens new opportunities for recycling using non-toxic liquids, but also bridges the gap between aqueous leachates employed in hydrometallurgy and organic solvents used in solvometallurgy.

### 2.4. Biohydrometallurgical Treatment

Biohydrometallurgical recovery of metals relies on chemical species generated during the metabolism of microorganisms such as bacteria and fungi. These metabolites, including various acids and redox-active compounds, can promote metal dissolution under mild and environmentally benign conditions. Despite the growing interest in bioleaching as a sustainable alternative to conventional hydrometallurgical routes, only a limited number of studies [113,114,115] have investigated its application to spent NdFeB magnets (Table 4). Existing laboratory research primarily explores the ability of selected microbial strains to mobilize rare earth elements from magnet powders, yet the reported data remain modest for larger operation scale.

Papajewski [113] compared the leaching potential of two bacterial species, *At. thiooxidans* and *At. ferriovans*, operating under acidic conditions (after three days the pH stabilized at about 3 for the first species and at 4.5–5 for the second). *At. thiooxidans* proved ineffective for REE bioleaching, even when the medium was supplemented with sulfur, since these bacteria produce only H_2_SO_4_ as the leaching agent. Much better performance was observed for *At. ferriovans*, which generates both H_2_SO_4_ and Fe^3+^ ions capable of promoting leaching. As a result, nearly 84% dysprosium was extracted from the sample (compared to 76% for Nd). Auerbach et al. [114] obtained similar dysprosium extraction efficiency (86%) using *At. ferrooxidans* for NdFeB powder (2% Dy). At the same time, the leaching efficiency decreased when the physical form of the magnets was changed to slices (1.2% Dy in magnet, 44% Dy leached) or blocks (6.2% Dy in magnet, 5% Dy leached). Further bioleaching carried out in a larger bioreactor (3.5 L) with a *L. ferrooxidans* strain showed high efficiency, enabling almost complete dysprosium extraction (99%), although Nd and Pr were recovered at lower levels (92% and 75%, respectively). The leach solution was subsequently purified by solvent extraction of REEs using Cyphos IL101 and DEHPA, followed by oxalate precipitation, which yielded a final product with 98% purity.

Magrini and Jagodzińska [115] conducted the first cost assessment of a complete biohydrometallurgical recycling process for NdFeB magnets. They evaluated six consecutive stages of the workflow: demagnetizing, shredding, bacterial cultivation, bioleaching, REE extraction and final oxidation, which produced a mixed oxide (87% Nd_2_O_3_, 6% Dy_2_O_3_ and 4% Pr_2_O_3_) with an overall 96% purity. For each stage, system, energy and material costs were estimated, taking into account material losses and assuming a maximum processing capacity of about 103 kg NdFeB magnets per month. Based on these calculations the net profit could be estimated at about 23% of the total operational costs. These findings indicate that, although research on green biohydrometallurgical approaches remains at a very early stage, the method shows promising potential for further optimization.

### 2.5. Solvometallurgical Treatment

Solvometallurgical methods employ organic solvents, including molecular liquids, ionic liquids, and deep eutectic solvents, to promote metal dissolution and enable selective recovery [116]. Molecular liquids are conventional non-aqueous media composed of polar organic compounds, both aprotic and protic. These solvents are inexpensive, readily available, and possess advantageous physicochemical properties such as low viscosity and good solubility for metal salts. In contrast, ionic liquids typically consist of large organic cations paired with smaller organic or inorganic anions. They exhibit a wide liquid range with melting points below 100 °C, high solubility for metal salts, and good chemical and thermal stability. However, their complex synthesis and purification procedures make them considerably more expensive. In turn, deep eutectic solvents are mixtures of two or more molecular compounds (a hydrogen-bond acceptor combined with a hydrogen-bond donor), with a melting point lower than that of the individual components. They show similar physicochemical properties with ionic liquids, including a wide liquid range, low volatility, and high solubility for both inorganic and organic compounds, but their simple preparation, achieved by mixing the components with moderate heating, makes them significantly easier and more cost-effective to produce. Nevertheless, they are more viscous than conventional aqueous and non-aqueous solvents, are not resistant to high temperatures, and may begin to decompose at around 100 °C. These organic systems have been explored less extensively than classical hydrometallurgical routes and have generally not yet been implemented due to technological and economic barriers [117]. Nevertheless, laboratory studies [118,119,120,121,122,123,124,125,126,127,128] often demonstrate preferential dysprosium than neodymium recovery, often with combination of hydrometallurgical approach (Table 5).

Önal et al. [118] proposed a three-step method for recovering metals from spent NdFeB magnets: (i) alkali baking with concentrated NaOH solution to convert the metals into hydroxides, (ii) leaching in an organic solution of Versatic Acid 10, and (iii) precipitation of oxalates from the organic leachate. Although the dysprosium concentration in the initial raw material was very low (0.14%), complete recovery was achieved.

A series of studies [119,120] focused on modifying solvent extraction by adjusting the composition of synthetic leachate solutions. This was achieved by gradually replacing water with polar organic molecules such as ethylene glycol EG [120], polyethylene glycol PEG200 [119,120], and dimethyl sulfoxide DMSO [120]. These compounds can weaken the hydration sphere of REE ions, thereby influencing their extraction and separation by organic extractants like Cyanex 923 [119] or P350 [120]. In all cases, preferential extraction of dysprosium over neodymium was obtained, with the best results observed for systems containing 60–80% organic modifier in the leachate solution. Against this background, PEG200 stands out in particular, as a Dy/Nd separation factor of up to 100 can be achieved (at 70 vol% PEG200). The extraction stage can be followed by stripping with HCl at much lower concentrations (0.4 M) than those typically used in conventional solvent extraction from aqueous media.

Melegari et al. [121] exploited the specific ability of chloride-substituted 8-hydroxyquinoline Q_Cl_ and tetrabutylammonium hydroxide Bu_4_NOH to form complexes of different solubilities in a synthetic nitrate–ethanol solution, enabling the separation of Dy^3+^ from Nd^3+^. In this system, the readily soluble dysprosium complex (Bu_4_N)[Dy(Q_Cl_)_4_] could be separated from the insoluble neodymium compound [Nd(Q_Cl_)_9_]. Dysprosium was subsequently recovered by precipitation with an aqueous oxalic acid solution, yielding a final product of about 99% purity.

Ionic liquids have been identified as highly effective media for the dissolution of milled NdFeB magnets (Table 5). Orifice et al. [122] investigated several roasting procedures, including high-temperature treatment in air or in the presence of CaCO_3_ flux and/or carbon. The optimal conditions combined the use of a carbon additive, which prevented the formation of the undesirable mixed oxide NdFeO_3_, with no quenching required. Subsequent leaching was performed in betainium bis(trifluoromethylsulfonyl)imide ([Hbet][Tf_2_N]), which exhibited high selectivity, enabling complete dissolution of dysprosium and neodymium while limiting iron dissolution to about 5%. The selectivity was further enhanced when the leaching solution contained 10 wt% water.

Other studies [123] examined a mixture of trihexyltetradecylphosphonium chlorides, [P_666_,_14_][Cl_3_] and [P_666_,_14_][Cl]. In this system, up to 100% of dysprosium could be dissolved, and the selectivity toward Dy over Nd and Fe increased slightly with temperature (from 25 to 70 °C). Interestingly, the subsequent stripping of REE ions (Dy, Nd, Pr) can be performed selectively using 3–5 M NaCl aqueous solutions, as the stripping efficiency for Co and Fe ions decreases at higher salt concentrations. Both transition metals can then be recovered in a second stripping step using ammonia solution, yielding a cobalt-containing solution and an Fe(OH)_3_ precipitate. This approach enables a nearly closed-loop use of the ionic liquid throughout the process.

Another report [124] showed the use of molten pyridine hydrochloride PyHCl as an ionic liquid leachant. It was found that NdFeB powder could be completely dissolved within 10 min at 165 °C without stirring. This stage was non-selective, as dysprosium, neodymium, and iron were all extracted. The selective separation of metal ions was carried out in three consecutive stages using different extractants: PC 88A in p-cymene for dysprosium, pure PC 88A for neodymium, and Cyphos IL101 in p-cymene for iron. The REE ions were then precipitated as oxalates, iron was recovered as a hydroxide, while PyHCl could be regenerated by treatment with HCl.

Recently, hydrophobic deep eutectic solvents have been extensively studied as potential mixtures for the extraction of REEs in the recycling process of NdFeB magnets [125,126,127]. In fact, these studies utilize synthetic solutions for liquid–liquid extraction, bypassing the leaching stage, which makes the crucial dissolution step somewhat unclear. Unfortunately, the reported systems, such as trioctylphosphine oxid with thymol TOPO + THY [125] or DEHPA + menthol [126], effectively separate REEs from cobalt and iron but do not selectively target dysprosium ions. Hanada et al. [127] investigated the kinetics of leaching metal oxides (iron, cobalt, neodymium, dysprosium) in benzoyltrifluoroacetone with trioctylphosphine oxide HBTA + TOPO and benzoyltrifluoroacetone with tributylphosphine oxide HBTA + TBPO mixtures. It was found that HBTA + TBPO is highly selective for the complete dissolution of Nd_2_O_3_ within a few minutes, while the addition of a small amount of water (2.5%) further facilitated the dissolution of Dy_2_O_3_ (100%) and CoO (80%). In contrast, HBTA/TOPO systems were highly selective for Nd_2_O_3_ dissolution only in the presence of water. Both organic systems were also tested for liquid–liquid separations, showing a high tendency to transfer ions from aqueous solutions, except for cobalt, at pH levels below 2.

## 3. Dysprosium Recovery from Phosphogypsum

### 3.1. General Characterization

Phosphogypsum is a major by-product generated in the production of phosphate fertilizers [128]. It forms during the digestion of raw fluoroapatite Ca_10_(PO_4_)_6_F_2_ with concentrated H_2_SO_4_, yielding phosphoric acid H_3_PO_4_, hydrogen fluoride HF, and calcium sulfate dihydrate CaSO_4_·2H_2_O. It is estimated that each tonne of H_3_PO_4_ produces roughly 3.5–5 tonnes of phosphogypsum [128,129], amounting to nearly to near 300 million tonnes of waste generated globally each year [129,130]. About 85% of this waste is stored near fertilizer plants in stockpiles or is discharged directly into aquatic environments [128,129,130,131,132] showing harmful impact [133,134]. Although phosphogypsum is not classified as hazardous waste, the presence of radioactive elements (up to 0.01%) or the release of toxic constituents (fluorine: 0.5–1.7%, heavy metals: below 1%) [128,129,130,135], may justify assigning it to a hazard-related category [136,137]. On the other hand, phosphogypsum contains relatively large amounts of rare earth elements (0.005–0.6 wt%) [130,138], making it a prospective secondary source for their recovery [130,132,138]. The dysprosium content in this waste varies widely, from a few to several tens of ppm (Table 6). Although it is noticeably lower than in primary sources (Table 1), this potential remains worth considering.

In recent time, a few reviews have addressed the hydrometallurgical recovery of REEs from phosphate rocks and phosphoric acid [155] pointing also phosphogypsum as a potential source [138,156,157,158,159]. However, the number of detailed studies on the latter mostly consider the general leachability behavior of the entire group of elements in a laboratory scale, only sometimes focusing on those with the highest percentage content, such as lanthanum, cerium, yttrium and neodymium. As a result, the end products are mainly considered concentrates containing several elements, with no further specification of their separation. While no commercial operations for rare earth recovery from phosphogypsum currently exist, this highlights both the technical and, most importantly, economic challenges of these processes [158,160]. Nevertheless, a few pilot-scale projects have been tested, such as those in Poland (leaching in H_2_SO_4_ followed by neutralization with ammonia to produce 10−15% REE primary concentrate [161]), Russia (leaching in H_2_SO_4_ followed by sorption on cation exchangers, neutralization with ammonia and precipitation with ammonium carbonate to produce REE concentrate [162]), or South Africa (leaching followed by continuous ion exchange and solvent extraction to produce a high-grade mixed rare earth product [163,164]).

Although the recovery of rare earths from phosphogypsum has recently gained attention, there is still a lack of detailed information on the performance of individual elements. This is particularly important in the case of dysprosium, which is the most critical element in the group. While (bio)hydrometallurgical methods have been examined in this context [139,140,141,142,143,144,145,146,147,148,149,150,151,152,153,154], solvometallurgical approaches have not reported the behavior of dysprosium [165,166].

### 3.2. Hydrometallurgical Treatment

Direct leaching of phosphogypsum involves the application of inorganic acids (Table 7). Since most rare earth element ions are hosted within the gypsum phase (with only 25% in monazite), primarily as sulfates, carbonates, fluorides, phosphates, or through adsorption [156], the low solubility of CaSO_4_ presents a significant barrier during extraction [159]:

xCaSO_4_·REE^3+^↓+ yH^+^ → zREE^3+^ + zH^+^ + zCa^2+^ + (x–z)CaSO_4_↓ (8)

Maina et al. [149] compared the leachability of REEs using a variety of leachants, including common acids at different concentrations (0.5–2 M), as well as acidic water (pH 3) and distilled water. They used the fraction with the smallest particle size (max 200 μm), which represented 50–75% of the total phosphogypsum powder (up to 800 μm). Dysprosium leachability in HNO_3_ and HCl was about 95%, but it dropped to about 82% for H_2_SO_4_ and around 60% for H_3_PO_4_ and acidic water. This behavior was similar to that observed for Gd, Tb, Ho, and Er, but not for other REEs, especially the hardly leachable ytterbium. Further studies [167] on acid leaching (2M H_2_SO_4_) showed a slightly better recovery for the smallest particle fraction (about 85% for 200 µm) compared to the three larger fractions (about 81% for 400–800 µm). Interestingly, leaching of Tm, Lu, and Yb was significantly more difficult from the larger particles than from the smallest fraction, with a difference of 20–30%. Although modifications to the waste sample (drying, calcination, prewashing with 20% Na_2_SO_4_) and leaching conditions (using oxidants or reducing agents) were made, no detailed results were presented. The only information provided was that for prewashed phosphogypsum with Na_2_SO_4_ (to increase sulfate ions, stabilize pH, and remove soluble impurities like organics, phosphates, or fluorides), total leachability of REEs decreased to about 73%.

**Table 7:** Examples of dysprosium recovery from waste phosphogypsum by hydrometallurgical treatment.

Dy Content in Waste, ppm	Pretreatment Stage	Leaching Conditions	Leaching Efficiency, %	Separation Methods	Dy Recovery, %	Final Product	Ref.
44 ± 2	sieving (≤200 μm)	1 or 2 M HNO_3_, 25 °C, S/L 8, 4 h	92–99	–	–	REE nitrate solution	[149]
		2 or 4 M HCl, 25 °C, S/L 8, 4 h	96–98			REE chloride solution	
		0.5–2 M H_2_SO_4_, 25 °C, S/L 8, 4 h	80–85			REE sulfate solution	
		2 M H_3_PO_4_, 25 °C, S/L 8, 4 h	61			REE phosphate sol.	
13	–	3 M HNO_3_, 25 °C, S/L 8, 8 h	85	–	–	REE nitrate solution	[151]
		0.5 M H_2_SO_4_, 25 °C, S/L 8, 8 h	49			REE sulfate solution	
12	–	0.5 M HCl, 25 °C, S/L 3%, 1 h	86	–	–	REE chloride solution	[141]
14	water washing, drying (80 °C)	3 M MSA, 25 °C, S/L 8, 2 h	72	–	–	REE MSA solution	[146]
		4 M PTSA. 25 °C, S/L 8, 2 h	75			REE PTSA solution	
		2 M HCl, 25 °C, S/L 8, 2 h	72			REE chloride solution	
16.6	NaCl washing (0.4 M, 10 min); Na_2_CO_3_ washing (0.6 M, 90 °C, 1 h)	1.5 M H_2_SO_4_, 100 °C, S/L 3%, 2 h	(55 mg/L)	precipitation with NH_3aq_	98	REE-rich (NH_4_)SO_4_ crystals	[153]
9.5	microwave (1200 W, 15 min)	1.5 M HCl, 85 °C, S/L 8, 1 h	99	–	–	REE chloride solution	[168]
4.4	milling (steel rod, 10 min)	PX–107	–	Chelok^®^Polymer /HNO_3_	85	REE nitrate solution	[154]

The leaching abilities of strong inorganic acids were also confirmed by other reports. Li et al. [141] identified HCl as the optimal leachant due to high recoveries of dysprosium and neodymium, correlating it with the occurrence modes of rare earths, such as Ca^2+^ substitutions or separate oxide or sulfate phases trapped inside phosphogypsum crystals. In turn, Cánovas et al. [151] showed better dysprosium leachability with HNO_3_ (85%) than with more diluted H_2_SO_4_ (49%). Interestingly, similar recovery levels were observed for Pr, Nd, Sm, Eu, Gd, Tb, Ho, and Er (46–52%) in H_2_SO_4_, while the differences between REEs practically disappeared in HNO_3_ (81–61% recovery. These studies led to the recommendation of using 0.5M H_2_SO_4_ for a washing step to produce an REE leach liquor for further concentration, while simultaneously preventing gypsum dissolution (only 6%).

Lütke et al. [139] applied ultrasound-assisted leaching in H_2_SO_4_, which improved REE leachability (by about 15% for dysprosium) by enabling the use of lower acid concentrations (0.6M) and reducing the leaching time (even to 20 min).

Brahim et al. [146] used unconventional acids as leachants, namely methanesulfonic acid MSA and p-toluenesulfonic acid PTSA, both regarded as eco-friendly reagents. Dysprosium recovery in both acids was 72–75%, comparable to that found for HCl solution under optimal leaching conditions. This behavior is similar to that observed for Er (70–74%) and Eu (60–68%), while for other REEs, significantly better results were obtained with MSA. Interestingly, the solubility of REE-MSA salts is intermediate between the most soluble chlorides and the least soluble REE-PTSA salts (notably, solubility decreases with increasing temperature in PTSA). However, according to the shrinking core model, leaching in both sulfonic-type acids is controlled by product layer diffusion, whereas in HCl, it is governed by chemical reaction.

Kuriken et al. [169] performed resin-in-leach (batch and column) experiments, where they agitated resin (Purolite S940) with phosphogypsum and sulfuric acid (25 °C, 20 h), followed by wet sieving. After water washing, the resin was reused in a new portion of the slurry. After eight cycles of resin loading, the concentration of REEs in the resin was estimated. The REEs grouped according to their loading as follows: 10^−1^ mol/kg (Ce), 10^−2^ mol/kg (Y, La, Pr, Nd, Sm), 10^−3^ mol/kg (Eu, Gd, Dy), 10^−4^ mol/kg (Sc, Tb, Ho, Er, Yb), and 10^−5^ mol/kg (Tm, Lu). Since the composition of the phosphogypsum was not provided, it is difficult to determine if any specific REE shows preferential adsorption on the resin. Various eluents (organic acids or their salts) were used to remove ions from the loaded resin, with the first elution step involving calcium and strontium removal using HCl. The second step, with an alkaline eluent, resulted in a concentrated REE solution as the final product.

**Figure 9:**
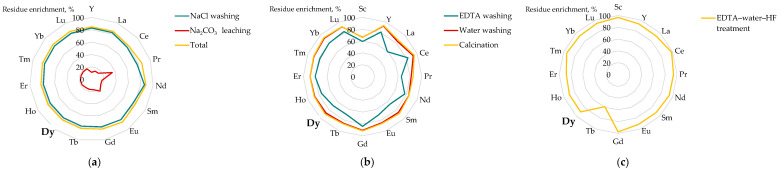
Effect pretreatment method on enrichment of REE in phosphogypsum residues: (**a**) subsequent washing with NaCl and Na_2_CO_3_ solutions (adapted from [152]), (**b**) subsequent washing with EDTA and water followed by calcination (700 °C, 3 h) (based on [170]); (**c**) washing with EDTA, water followed by HF treatment (based on [170]).

A series of investigations proposed different strategies of phosphogypsum pretreatment before leaching. Hammas-Nasri et al. [152] reported a two-step treatment of the waste by washing with NaCl solution followed by less effective leaching with Na_2_CO_3_ solution (Figure 9a). This process removed soluble impurities, gradually concentrating REEs in the solid phase up to 84%. The obtained concentrate was then leached in a two-step process with H_2_SO_4_ in a high-pressure reactor [153], producing an REE-rich liquor (4.3 g/L). Sequential element recovery was then performed through ammonia recovery (in 3 stages up to pH 6), with nearly 99% of the REEs incorporated into ammonium sulfate crystals.

Chanuri et al. [170] compared two beneficiation methods based on sequential treatment of raw phosphogypsum with 0.4 M EDTA, distilled water, followed by calcination (Figure 9b), or HF treatment (Figure 9c). This resulted in a concentration of REEs by a factor of 18.4, from 0.2–0.4 wt% up to about 4.4 wt% (90% Dy enrichment). The treatments led to a gradual change in the phase composition of the solid residues; however, no further leaching was carried out to assess the effectiveness of the pretreatment.

**Figure 10:**
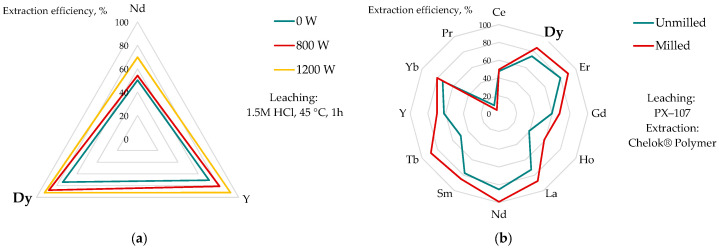
Effect pretreatment method on extraction of REE from phosphogypsum: (**a**) microwave treatment for 15 min (based on [168]); (**b**) milling for 10 min (based on [154]).

Lambert et al. [168] used microwave treatments followed by acid leaching (1.5 M HCl) to recover dysprosium, neodymium, and yttrium. They found improved dissolution efficiency at moderate temperatures with increased microwave power (Figure 10b). However, practically complete dissolution of Dy and Y was achieved only at a high leaching temperature (85 °C). Under these conditions, a maximum of 80% of neodymium could be only extracted. The application of microwave treatment enhanced the infiltration of lixiviant ions into the pores and cracks, but also altered the phase composition of phosphogypsum, from the dominant gypsum CaSO_4_·2H_2_O (near 80%) to hemihydrate CaSO_4_·0.5H_2_O with anhydrite CaSO_4_ (50:50).

A certain method for REE recovery was proposed in the project report [154]. Milled and unmilled phosphogypsum was leached in a patented PX-107 solution, followed by REE ion extraction with Chelok^®^ polymer (poly(1-octadecene-2,5-furandione salt)), and elution with HNO_3_ (Figure 10b). It was found that most of the REEs were recovered at yields above 80% (85% for Dy) from milled waste. Milling itself improved leaching efficiency by an average of 11%, although this increase could be very low (e.g., 1.8% for Ce, 7.3% for Pr) or significant (e.g., 20% for Ho and 39% for Tb). Although the method seems quite simple and cost-effective, it requires pH regulation before adsorption (pH 1.5) and cannot be applied for recovery from phosphoric acid.

Hydrometallurgical pretreatment and leaching of phosphogypsum result in effective REE recovery, including dysprosium. Although, the behavior of individual elements is not uniform, as it is highly dependent on the nature of the reagents used, dysprosium dissolution is at medium to high levels, mostly above 70%.

### 3.3. Biohydrometallurgical Treatment

Bioleaching of phosphogypsum has been explored for REE recovery [170,171,172,173,174,175,176], but the performance of dysprosium is often overlooked in reports [170,171,172]. Recent studies have investigated the use of biological sulfur-based media for treating phosphogypsum and its leachate. For example, Saolo et al. [173,174] used *Desulfovibrio* bacteria to reduce sulfate to sulfide in an anaerobic environment within a bioreactor, preceded by water or sulfuric acid leaching. While REE extraction with water remained below 1%, a 25% recovery (15% for Dy) was achieved with diluted acid (0.02 M). Bioreactor treatment resulted in 97% sulfur and over 99% REE removal, though the tolerated acid concentration was limited to 0.01 M. The bioreactor precipitate accumulated lanthanides making it suitable for further recovery. This material contained 47 ppm of dysprosium, primarily dispersed across all identified phases.

In turn, Tayar et al. [175] compared the bioleaching behavior of sulfur-oxidizing bacteria from acid mine drainage and *At. thiooxidans*. Since the latter produced more sulfuric acid, it was more extensively examined for phosphogypsum treatment. The highest REE extraction (60%) was obtained through two-step bioleaching, and at the reactor scale (3L S/L 10%), total REE recovery reached about 55%. Among all REEs, neodymium was the most easily leached (98%), while the leachability of other elements, including dysprosium, was around 60%. The released REEs were subsequently recovered as a concentrate through precipitation as oxalates.

**Figure 11:**
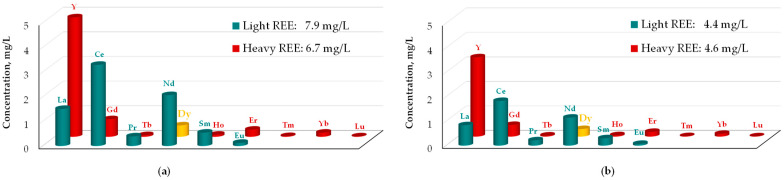
Effect leaching mode on extraction of REE from phosphogypsum: (**a**) bioleaching with *Aspergillus niger*; (**b**) chemical leaching with a mixture of organic acids with a composition similar to fermentation liquid (based on [176]).

It is worth noting that, compared to chemical acid leaching, bioleaching utilizes metabolites produced by microorganisms. Apart from biogenerated sulfuric acid, organic acids produced by fungi could show high potential due to their complexing properties; however, these have not been exploited intensively for phospogypsum. Zhang et al. [176] analyzed the extraction of rare earth elements using *Aspergillus niger*, which produces citric, gluconic, oxalic, tartaric, and ketoglucaric acids. They found that bioleaching significantly improves the leachability of elements, but heavy rare earth elements were leached less efficiently than light REEs when compared to chemical leaching with an organic acid mixture (Figure 11). Although the leaching efficiencies of individual elements were not provided, the data indicate a high potential for utilization.

## 4. Dysprosium Recovery from Coal Combustion Ashes

### 4.1. General Characterization

The combustion of coal in power plants generates two main waste streams: fly ash (40–90%) and bottom ash (10–20%) [177]. Fly ash is a fine powder (up to about 200 μm) captured from flue gases through electrostatic or mechanical precipitation. It is estimated that for every four tons of coal burned, one ton of fly ash is generated, totaling 600–800 million tons annually worldwide [178]. In contrast, bottom ash [177] consists of agglomerated, porous particles (millimeter size range) that are too large to be carried into the flue gases and therefore accumulate at the bottom of the coal furnace. The global annual production of coal bottom ash is estimated at 780 million tons, with the majority coming from Asian countries, followed by contributions from Europe and the USA [179]. Although both types of ash can be reused, a large percentage is still stored in landfills and ash ponds [178,179,180].

The main components of coal ashes are (alumino)silicates and metal oxides (iron, calcium, magnesium, etc.), but in varying proportions. Since they often contain valuable elements, they can also serve as a significant source of rare earth elements [180,181]. The primary source of metallic elements in both types of ash is coal. Ketris and Yudovich [182] estimated that the mean dysprosium concentrations in world coals are similar, i.e., 2.0 ± 0.1 ppm in brown (lignite) coal and 2.1 ± 0.1 ppm in hard (bituminous) coal. However, during combustion, the coal material oxidizes, increasing the concentration of the element to 12 ± 1 ppm in brown coal ash and 15 ± 1 ppm in hard coal ash. However, the actual concentrations of elements in coal ashes from power plants vary widely [183,184,185,186,187,188,189,190,191,192,193,194,195,196,197,198,199,200,201,202,203,204,205,206,207,208,209,210], depending not only on the type of coal used but also on its regional origin (Table 8).

A comparison of dysprosium contents in both types of coal ash originating from the same power plant source shows quite similar concentrations [192,205,209,211], less often higher or lower [206,208,211]. Interestingly, in most cases, the proportion of dysprosium among all REEs (130–1700 ppm [195]) in coal fly ash remains at a level of 2.2–2.8%, although in some cases it can reach as high as 12% (e.g., in Nigerian fly ash [187]). For coal bottom ash, REE concentrations are almost in the same range (140–1000 ppm [195]), so dysprosium can achieve a similar share among the group. This low proportion should not be surprising, as the light rare earth element subgroup constitutes a majority of the total REE content in coal ashes, typically of order 70–80% [203,207,208,211]. Higher concentrations of dysprosium are also observed in finer particle fractions, below about 45 µm [199,206,210,211,212,213], which can be utilized in physical separation enrichment [181,199,214].

Dysprosium in coal ashes is associated with different modes of occurrence. In the fly ash, it is primarily found in the aluminosilicate fraction (48–80%), while organic/sulfide (7–47%), metal oxides (4–6%), and acid-soluble (about 1%) forms constitute the remainder [192,196,201,205,212,213]. A similar distribution is observed in bottom ash [192,205]. This corresponds to the accumulation of dysprosium in the nonmagnetic phase (60–65%) [192,215]. The chemical form of dysprosium influences the selection of reagents for its subsequent treatment with aqueous solutions and biologically generated media.

### 4.2. Hydrometallurgical Treatment

Hydrometallurgical methods for REE recovery from coal ash have recently garnered significant attention, leading to a few reviews for last year [216,217,218]. However, due to the complexity of the material, the entire REE group is typically considered as a whole, without a detailed focus on individual elements. Nevertheless, the distinct behavior of dysprosium can be traced in some studies [201,202,203,212,219].

Choudhary et al. [219] employed water and various organic acids and salts (e.g., ascorbic acid, acetic acid, oxalic acid, ammonium oxalate, hydroxylammonium chloride, ammonium sulfate, and some mixtures of these) for sequential extraction. Despite using different operational conditions (time, pH, concentration), dysprosium remained relatively stable in solid residues at a consistent level (12–17 ppm), owing to its accumulation in the nonreactive glassy aluminosilicate phase. Treatment with NaOH solution enhanced the dysprosium content in coal fly ash particles by 39% (from 13 ppm to 22 ppm Dy), while other solutions like KOH, HCl, and oxalic acid were less effective, resulting in an increase in dysprosium concentration by only 10–24%. Thus, alkaline pretreatment of glassy matrix with NaOH:

6NaOH + 3Al_2_O_3_·2SiO_2_↓→ 2NaAlSiO_4_ + 4NaAlO_2_ + 3H_2_O (9)

appeared to be a promising chemical beneficiation method, also showing optimal results for other REEs.

Pan et al. [212] combined NaOH treatment of fly ash with subsequent leaching using citric acid. This alkaline hydrothermal method doubled the dysprosium (and other REE) concentration in the solid phase (up to 51 ppm) by removing silicon and aluminum components (by 60–75%). This resulted in a 55% efficiency in REE extraction, although a comparison with the values for the non-pretreated sample was not provided.

Other studies have focused on direct leaching of coal ashes. Among the leaching agents tested (Table 9), only more concentrated (2–4 M) HCl acid solutions at elevated temperatures (80 °C) were able to leach a maximum of 53–55% of dysprosium [202,203]. Interestingly, the leaching results were worse than those for discarded coal (4.8 ppm Dy, 65–75% recovery). The resulting solutions contain only trace amounts of dysprosium ions, requiring advanced recovery methods, as similar chemical behavior among the group elements is observed. Unfortunately, most scientific studies conclude at this stage, without providing answers for the subsequent selective separation of metal ions.

### 4.3. Biohydrometallurgical Treatment

Microbiological leaching leverages the diverse capabilities of species to release metals from coal ashes. Chaerun et al. [222] utilized the mixotrophic bacterium *Alicyclobacillus ferrooxydans*, an iron-oxidizing species capable of both autotrophic and heterotrophic growth. This bacterium secretes bio-organic metabolites that accelerate mineral dissolution through acidolysis and complexation processes. However, these species (even when supplemented with pyrite) were only able to selectively extract gadolinium, lutetium, and terbium, with no significant effect on dysprosium release from fly ash (max. 1–3% recovery).

**Figure 12:**
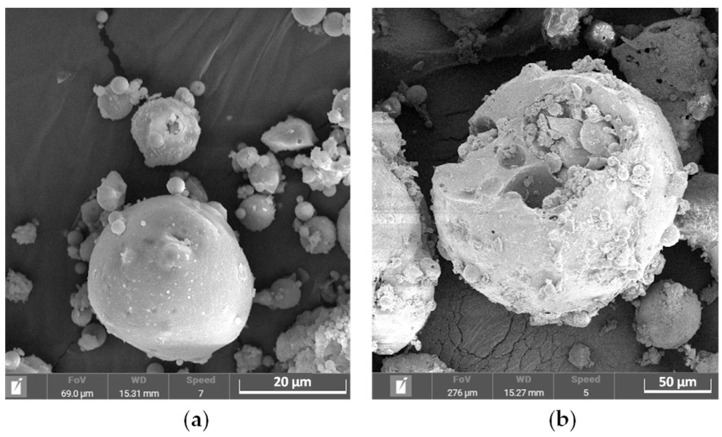
SEM micrographs of pulverized coal furnace coal fly ash before (**a**) and after bioleaching (**b**) with silicate bacterium *P. mucilaginosus.* Adapted from [223] under License CC BY 4.0.

Another approach was employed by Shi et al. [223] to remove the glassy matrix that hinders the leaching of valuable metals. They used the silicate bacterium *Paenibacillus mucilaginosus* to convert insoluble silicon into a bioavailable form by secreting organic acids (oxalic, tartaric, citric, and malic). The biological pretreatment of the waste enhanced the material’s porosity (Figure 12), increasing the reaction area and facilitating leachant access. This improved the subsequent chemical acid leaching (1–3 M HCl) efficiencies by 5–15% on average, with dysprosium recoveries reaching up to 40%. Comparable results for the bioleaching of dysprosium can be achieved using the fungus *Aspergillus niger* [224]. Leachability was pH-dependent, with nearly 40% recovery under optimal conditions (pH 2, 28 °C, 14 days).

It is noteworthy that bioleaching appears to be a promising method, yielding comparable results to chemical leaching of coal ashes. However, the recovery of dysprosium (and other heavy REEs) is generally lower than that of other lanthanides.

### 4.4. Solvometallurgical Treatment

**Figure 13:**
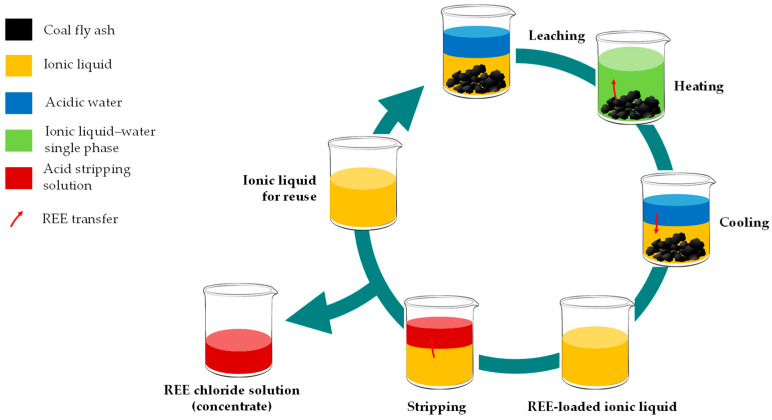
Closed-loop recovery of REE from coal fly ash using [Hbet][Tf_2_N] ionic liquid. Based on [226,227].

The application of organic solvents for dysprosium recovery from coal ashes is still in its early stages, despite numerous examples of ionic liquids being used for REE recovery from secondary sources [225]. Betainium bis(trifluoromethylsulfonyl)imide [Hbet][Tf_2_N] appears to be the most extensively studied ionic liquid in this area [226,227,228]. It demonstrates unique behavior, such as an increase in mutual solubility with water as temperature rises, eventually leading to a single-phase system (above 55 °C), along with excellent solubility for REOs. This effect has been utilized for coal ash leaching, combining leaching and stripping procedures in a closed-loop cycles (Figure 13). The leaching effects of [Hbet][Tf_2_N] were found to be dependent on the origin of coal fly ash, with the leaching efficiency increasing for specific materials in the following order: unweathered ash Class-F (19 ppm) < weathered ash Class-F (from ash pond; 14 ppm Dy) < unweathered ash Class-C (fresh from the power plant; 7 ppm Dy) [226]. Specifically, the respective dysprosium recoveries were 20%, 40%, and 84%. Pretreating fly ashes with NaOH improved leachability, with dysprosium recoveries ranging from 80 to 100, with higher concentrations of [Hbet][Tf_2_N], the leachability of some REEs reached 100%, while the extraction of major elements (Si, Al, Fe, Ca) was at 20–80%. Reuse experiments with the ionic liquid over three rounds confirmed its stable properties, with no significant change in characteristics and maintaining a high dysprosium extraction capacity (about 85%).

Further studies [227] showed that changes in the pH of the aqueous phase (2–7), time (0.5–12 h), or temperature (45–85 °C) had little impact on extraction efficiency, with dysprosium recovery remaining at approximately 55% (for unweathered Class-F ash). Optimization of the procedure [228] by introducing additional steps—cooling the leaching liquor, adding betaine and ascorbic acid, and reheating to 85 °C during the leaching stage, along with incorporating an intermediate heating step (to 85 °C) in the stripping stage—resulted in improvements in the process. Metals such as Sc, Y, Nd, Sm, Gd, Dy, and Yb consistently showed high leaching and partitioning into the organic phase, with average recovery efficiencies ranging from 54% to 66%. However, other REEs exhibited greater variability across the different coal fly ash samples.

## 5. Conclusions

A review of the literature on dysprosium recovery from various secondary materials underscores the essential role of this rare earth element, particularly for clean energy technologies, while also seeming to strongly confirm the origin of the element’s name. Despite the widespread availability of different waste materials in various regions, recovering dysprosium—one of the most critical rare earth elements—remains a major challenge. The main difficulty lies in the trace concentrations of dysprosium and the complex, multi-component nature of the materials. Among these, spent NdFeB magnets show the most promise for solvent-based recycling. In contrast, coal fly ashes, although widely available, bind dysprosium in an inert, unreactive phase, requiring substantial pretreatment to enhance the release of the desired element. Phosphogypsum, while not yet a significant source of dysprosium, is increasingly recognized as a potential source for other REEs (Table 10).

**Figure 14:**
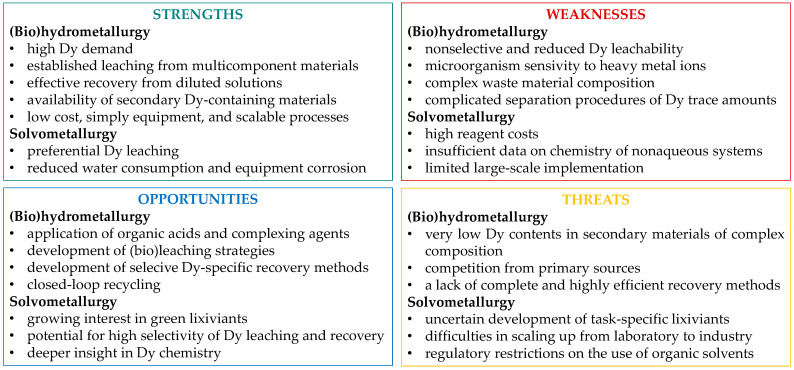
SWOT analysis of dysprosium recovery from secondary materials.

The initial stages of research appear promising, but transitioning from laboratory-scale experiments to large-scale, long-term industrial practices remains a significant hurdle (Figure 14). Traditional hydrometallurgical methods have been widely tested, but more innovative water-organic systems, blending hydro- and solvometallurgy, are emerging as promising alternatives. Ionic liquids, in particular, show potential for enhancing dysprosium dissolution; however, their industrial application remains limited, and they have not yet achieved widespread adoption.

These factors also open up opportunities stimulating scientific advancement and the development of innovative solutions to complex recovery problems. More efficient pretreatment methods and the optimization of extraction techniques could improve dysprosium recovery from secondary sources. Moreover, developing new materials for selective separation in solutions, capable of isolating dysprosium from other ionic impurities, could enhance the efficiency of the process. By refining these methods, there is potential to create more sustainable and cost-effective processes, ultimately leading to greater scalability and industry adoption.

## Figures and Tables

**Table 1:** Dysprosium concentration in primary rare-earth sources worldwide.

Country	Mineral Type	Dy_2_O_3_ in Total REOs, %	Ref.
China	bastnäsite, xenotime, rare earth laterite	0.1–9.1	[29,30]
India	monazite	0.18–0.24	[28,30]
Malaysia	xenotime, ion-adsorption clay *	8.3–8.44 (0.006–0.009) *	[29,31,32]
Myanmar	monazite	4.0	[33]
Vietnam	carbonatite, barite-fluorite *	0.2 (0.004–0.012) *	[28,33]
Australia	xenotime, monazite, trachyte, ionic clay	0.2–2.6	[28,29,30,34]
Canada	monazite, bastnäsite, fergusonite-(Y), apatite, eudialyte	0.35–4.1	[28,34]
United States	monazite, bastnäsite,	0.03–1.4	[29,31,34]
Brazil	monazite, alkaline granite	0.4–8.1	[28,29]
Madagascar	allanite, ion-adsorption clay *	0.08–0.17 (0.013) *	[32,35]
Namibia	carbonatite	(0.007–0.014) *	[36]
South Africa	monazite	0.7–0.8	[34]
Uganda	ionic clays	2.7	[28]
Greenland	steenstrupine-(Ce), eudialyte, alkaline rock	1.1–2.9	[28,34]
Norway	carbonatite	0.31	[28]
Russia	loparite, carbonatite, alkaline granite	0.6–7.9	[28,30]
Sweden	eudialyte, alkaline rock	0.03–4.3	[28,37,38]

* Dy concentration, %.

**Table 2:** General characterization of neodymium permanent magnets. Adapted from [20,21,47,68,70,74,75,76,77,78].

NdFeB Source	Average Magnet Content	Dysprosium Content, wt%	Other Metals
Wind Turbines	80–850 kg/MW	2–8 (2–22 kg/MW)	Magnets *: Nd, Pr, Tb, Fe, Nb, Co
Electric Vehicles	1.5–5 kg/unit	2.3–5	
Electric Bikes	0.3–0.35 kg/unit	3–4	
Hard Disc Drives	3–20 g/unit	0.1–1.4	Coatings: Cu, Ni, Cr, Zn, Al, Au, Ag
Mobile Phones	0.96–1.5 g/unit	0.8–2	
MRI machine	1300–2500 kg/unit	1.2	

* Main components: 22–32% Nd, 53–68% Fe, 1–2% B, 0.1–13% Pr, up to 6.4% Ni, 0.5–4.2% Co.

**Table 3:** Examples of dysprosium recovery from waste NdFeB magnets by hydrometallurgical treatment.

Dy Content in NdFeB, wt%	Pretreatment Stage	Leaching Conditions	Leaching Efficiency, %	Separation Methods	Dy Recovery, %	Final Product	Ref.
Leaching in Inorganic Acids							
1.1	grinding (75−106 μm)	0.5 M HCl, 90 °C, S/L 3%, 2 h	~100	SX: Cyanex 302 /0.08vol% H_2_SO_4_	98	Dy sulfate solution	[92]
2.1	demagnetization (350 °C); grinding (<100 μm); roasting (CaCl_2_, 600 °C, 1.5 h)	(water leaching) 0.5 M HCl, 90 °C, S/L 10, 3 h	88	Precipitation with (COOH)_2_, 60 °C, 0.25 h	no data	4.9% Dy_2_O_3_ in REOs *	[88]
3.95	no data	5 M HCl, 25 °C, S/L 7%, 24 h	(0.9 g/L)	PC-88A impregnated resin/0.05−2 M HCl	~100	99% DyCl_3_ (solution)	[93]
3.2	demagnetization (300−800 °C); grinding (<500 μm)	1 M HNO_3_, 70 °C, S/L 1%, 2 h	~100	SX: TOPO−3M HCl	90	Nd, Dy chloride solution	[89]
1.3–4.6 (mixture)	no data	2 M H_2_SO_4_, 80 °C, S/L 12%, 2 h	(1.3 g/L)	SX: HDEHPA in dodecane /4−5 M H_2_SO_4_; Precipitation with (COOH)_2_	4.1 g/L	99% Dy_2_O_3_ *	[91]
Leaching in Organic Acids							
1.2	demagnetization (350 °C); grinding (<100 μm)	5–10 M CH_3_COOH, 60 °C S/L 1−3%, 24 h	90−97	none	no data	Nd, Dy, Co acetate solution	[84]
2.8	demagnetization; grinding	1.45 M C_6_H_8_O_7_, S/L 10%, 3 h	92 (1.98 g/L)	SX: D2EHPA in n-heptane or kerosene; Precipitation with (COOH)_2_	~100	8% Dy_2_O_3_ in REOs *	[86,87]
1.1	roasting (air, 400 °C)	1 M CH_3_COOH, 25 °C, S/L 1.25−3.3%, 24 h	~100	SX: D2EHPA	no data	no data	[95]
		1 M C_6_H_8_O_7_, 25 °C, S/L 1.25−3.3%, 24 h	~100				
Leaching in Water							
0.4	demagnetization (310 °C); grinding (<149 μm); roasting (NH_4_Cl, 300 °C, 3 h)	H_2_O, 95 °C, S/L 10%, 1 h	15−100 (0.35 g/L)	Precipitation with (COOH)_2_	no data	1.2% Dy in REOs *	[94]
6.3	ball milling (<40 μm); roasting (12–16 M H_2_SO_4_, 650 °C, 3 h)	H_2_O, 25 °C, S/L 2%, 24 h	68−100	none	none	REE sulfate solution	[96]

* Calcination at 500−800 °C to convert oxalate to oxide.

**Table 4:** Bioleaching of dysprosium from NdFeB permanent magnets.

Dy Content in NdFeB, wt%	Pretreatment Stage	Bioleaching Conditions	Leaching Efficiency, %	Ref.
5.8	demagnetization (400 °C); grinding (<250 μm)	*At. thioxidans*, S/L 1%, 14 days	0.4	[113]
		*At. ferrivorans*, S/L 1%, 14 days	84	
2.0	no data	*At. ferrooxidans*, S/L 1%, 14 days	86	[114]
		*L. ferrooxidans*, S/L 1%, 14 days	99	

**Table 5:** Examples of dysprosium recovery from waste NdFeB magnets by solvometallurgical treatment.

Dy Content in NdFeB, wt%	Pretreatment Stage	Leaching Conditions *	Leaching Efficiency, %	Separation Methods	Dy (Nd) Recovery, %	Final Product	Ref.
Molecular Liquids							
0.14	alkali baking: (40% NaOH, 200 °C, 1.5 h)	Versatic Acid 10 in Shellsol G70, S/L 5%, 60 °C, 1 h	95.9	Precipitation with (COOH)_2_, 25 °C, 2 h	~100 (95)	0.6% Dy_2_O_3_ in REOs	[118]
−	−	synthetic solution: PEG200−Nd−Dy−chloride	(4 g/L)	SX: Cyanex 923 in GS190 + decanol	~100 (100)	−	[119]
−	−	synthetic solution: DMSO−Nd−Dy−chloride	(4 g/L)	SX: P 350 in kerosene	55 (10)	−	[120]
−	−	synthetic solution: EG−Nd−Dy−chloride			55 (10)	−	
−	−	synthetic solution: PEG200−Nd−Dy−chloride			70 (10)	−	
−	−	synthetic solution: nitrate-ethanol		8-hydroxyquinoline + Bu_4_NOH	63	99%	[121]
Ionic Liquids							
8.4	milling; roasting (5% C, 1400 °C, 2 h)	[Hbet][Tf_2_N] + 10 wt% H_2_O, S/L 2%, 80 °C, 1 h	100	−	−	−	[122]
5.7	milling (<125 μm)	[P_666,14_][Cl_3_] + [P_666,14_][Cl] (1:1), S/L 5%, 70 °C, 24 h	100	3M NaCl	100	−	[123]
6.9	milling (<45 μm)	PyHCl, S/L 1%, 165 °C, 1 h	100 (11.5 g/L)	SX: PC 88A in p-cymene	100	−	[124]
Deep Eutectic Solvents							
−	−	synthetic solution: Nd−Dy–Fe–HNO_3_	(0.004 g/L)	SX: TOPO + THY /2M H_2_SO_4_	100 (85)	Dy, Nd sulfate solution	[125]
−	−	synthetic solution: Nd−Dy–Fe–HNO_3_	(0.02 g/L)	SX: DEHPA-menthol /5M HNO_3_	89 (93)	Dy, Nd nitrate solution	[126]
−	mixture of oxides	HBTA + TBPO, 24 h	~5	−	~5 (100)	−	[127]
−	−	synthetic solution: Nd−Dy−Co–Fe–(NH_4_)_2_SO_4_	(0.2 g/L)	SX: HBTA + TBPO	95 (95)	−	

* Symbols of compounds are explained in the further text.

**Table 6:** Dysprosium concentration in phosphogypsum waste.

Source Region	Dysprosium Content, ppm *	Ref.
Brazil (Santa Catarina)	30.1 ± 1.8	[139]
Canada (Alberta)	5/11.7	[140,141]
China (Yunnan)	7	[142]
Egypt (Abu Tartur)	3	[143]
Finland (Yara Siilijärvi)	13.4	[144]
Morocco (Ouled Abdoun, Gantour, Jorf Lasfar)	6–14	[145,146,147]
Philippines (fertilizer plant ponds)	7.6 ± 4.4	[148]
Poland (Wizów)	42–46	[132,149]
South Africa (Phalaborwa)	26.6	[150]
Spain (Huelva)	13	[151]
Tunisia (Sfax)	8.9/16.6	[152,153]
USA (Florida)	4.4	[154]

* 1 ppm = 0.0001%.

**Table 8:** Dysprosium concentration (in ppm) in coal fly and bottom ashes.

Source Region	Coal Type	Fly Ash	Bottom Ash	Ref.
Australia	brown or no data	0.7–49.6	5.6–29.7	[201,211]
Canada	no data	2.6	2.5	[205]
China	no data	10–21	8.8–12	[192,196,197,206]
Czech Republic	no data	7.8	–	[204]
Greece	lignite	4.7–5.8	–	[193]
India	lignite	6.9–42.3	2.8	[199,202,207]
Indonesia	no data	3.7–7.8	0.3–8.2	[209,210]
South Korea	no data, anthracite	3–8.9	1.4	[188,200]
Nigeria	no data	2–134	–	[187]
Poland	bituminous, lignite	8.1–16.7	5.6–8.9	[184,185,208]
Russia	no data	(5.9–16.5) *	–	[194]
South Africa	no data	6.7/13.8	4.1	[195,203]
Turkey	lignite	2.9–6.4	2.4–4.9	[190,191]
Ukraine	bituminous, anthracite	3.8–8.7	–	[198]
United Kingdom	semi-anthracitic	11.1	–	[184]
USA	bituminous	7.5–21	–	[186,189]

* In coal ash.

**Table 9:** Dysprosium recovery from coal ashes using hydrometallurgical treatment.

Dy Content, ppm	Leaching Conditions	Leaching Efficiency, %	Recovery Method	Recovery Effects	Ref.
Fly Ash					
9.2	0.1 M HCl, 25 °C, S/L 5%, 16 h	37	–	–	[201]
13.8	1–2 M HCl, 25 °C, S/L 10%, 2 h	50–53	–	–	[203]
no data	4 M HCl, 75 °C, S/L 25%, 24 h	(0.78 ppm)	TOGA-impregnated organosilica adsorbent	~73% adsorbed	[220]
	5 M HNO_3_, 75 °C, S/L 17%, 24 h	no data		~50% adsorbed	
no data	0.1 M HNO_3_, min 12 h *	(0.27 ppm)	carboxylated mesoporous carbon adsorbent	85–100% adsorbed	[221]
Bottom Ash					
2.8	4 M HCl, 75 °C, S/L 10%, 2 h	55	–	–	[202]
	4 M HNO_3_, 75 °C, S/L 10%, 2 h	38			
	4 M H_2_SO_4_, 75 °C, S/L 10%, 2 h	23			
	4 M FeCl_3_, 75 °C, S/L 10%, 2 h	0			

* Commercial coal fly ash roasted with NaOH (400 °C, 0.5 h) before leaching.

**Table 10:** Assessment of dysprosium recovery strategies from waste materials (Notation: + + +—high; + +—average; +—low; ?—unknown).

Aspect	Method	NdFeB Magnets	Phosphogypsum	Coal Ashes
Dy Concentration	–	+ + +	+	+
Dy Leachability	(Bio)hydrometallurgy	+ +	+ +	+
	Solvometallurgy	+ + +	+ +	+ +
Dy Leaching Selectivity	(Bio)hydrometallurgy	+	+	+
	Solvometallurgy	+ +	?	+ +
Dy Recovery Procedures	(Bio)hydrometallurgy	+ +	+	+
	Solvometallurgy	+	+	+

## Data Availability

No new data were created.

## Abbreviations

The following abbreviations are used in this manuscript:

IX	Ion-exchange
SX	Solvent extraction
SIR	Solvent-impregnated resin
CAGR	Compound annual growth rate
MRI	Magnetic resonance imaging
ICE	Internal combustion engine
REE	Rare-earth elements
REO	Rare-earth oxide
HREE	Heavy rare-earth elements
LREE	Light rare-earth elements
TREO	Total rare-earth oxides
